# Combination radioimmunotherapy with adoptive NK transfer targets cancer stem cells in canine models of bone and soft tissue sarcoma

**DOI:** 10.1186/2051-1426-3-S2-P4

**Published:** 2015-11-04

**Authors:** Robert Canter, Steven K Grossenbacher, Jiwon Sarah Park, Jaime Modiano, Erik Ames, Stephanie Mac, Arta M Monjazeb, Michael Kent, William Culp, Mingyi Chen, William Murphy

**Affiliations:** 1 University of California, Davis, Sacramento, CA, USA; 2 University of Minnesota, Minneapolis, MN, USA; 3 UC Davis Comprehensive Cancer Center, Sacramento, CA, USA; 4 UC Davis School of Veterinary Medicine, Davis, CA, USA

## Background

We have previously shown that NK cells preferentially target cancer stem cells (CSCs) in diverse human solid malignancies and that radiotherapy (RT) enhances NK targeting of CSCs in an NKG2D-dependent manner. We hypothesized that dog PBMC-derived NK cells could be similarly expanded and activated ex vivo for combination radioimmunotherapy in canine models of sarcoma.

## Methods

Dog NK cells were isolated from fresh PBMCs using Ficoll separation and CD5 depletion. Isolated NK cells (CD3+, CD5dim, TCR-) were expanded via co-culture with irradiated (100Gy) K562-C9-mIL-21 for 2-3 weeks in 100IU/mL recombinant human IL-2. Canine osteosarcoma (OSCA) tumor lines and fresh canine primary sarcomas were evaluated for susceptibility to NK killing before/after RT *in vitro* and in xenograft experiments with NSG mice. NK cytotoxicity was assessed in 4-16 hour killing assays by Flow cytometry using a BD Fortessa cell sorter (BD Biosciences) with 7-Aminoactinomycin as cell viability marker.

## Results

NK expansion was successful in 14/20 donors (including 9 tumor-bearing dogs) from baseline 4.5(±1.9) ×10^6^ cells to 103.5(±29.1) ×10^6^, mean increase 23.2X (±2.3). Canine NK cells were also responsive to human cytokines (IL-2, IL-12, and IL-18), but expansions were lower (1.6-3.5 fold expansion over 14 days). NK cytotoxicity to OSCA78, OSCA23, and NK-sensitive CTAC cells *in vitro* increased in a dose-dependent fashion reaching 74 – 88% cytolysis at effector:target ratios of 10:1 – 20:1 (P < 0.001). RT augmented NK cytotoxicity with greatest synergy at 2.5-5 Gy RT in 4-hour killing assays (1.3-3.4X increased killing, P < 0.01). At doses of 10 Gy and/or16-hour killing assays, only minor differences in overall killing were observed. Similar results were observed with RT sensitization to NK killing in primary canine sarcomas. In a dog sarcoma PDX model using focal RT, intravenous NK transfer, and hydrodynamic human IL-15 for *in vivo* NK support, focal RT increased NK homing to tumors by 3.8X±0.3 (P < 0.001).

## Conclusions

RT enhances NK homing and killing in canine models of STS. Dog STS appears to be a valuable model to facilitate clinical translation of NK radioimmunotherapy.

